# Harnessing Country Experiences for Health Benefit Package Design: Evidence-Informed Deliberative Processes and Experiences From the Joint Learning Network: Comment on "Evidence-Informed Deliberative Processes for Health Benefit Package Design – Part II: A Practical Guide"

**DOI:** 10.34172/ijhpm.2023.7856

**Published:** 2023-10-28

**Authors:** Somil Nagpal, Naina Ahluwalia, Lauren Oliveira Hashiguchi, Kathleen McGee, Martin Lutalo

**Affiliations:** ^1^The World Bank, Jakarta, Indonesia.; ^2^The World Bank, Washington, DC, USA.

**Keywords:** Peer-to-Peer Learning, HTA, Evidence-Informed Deliberative Processes, Joint Learning Network, UHC, Systematic Priority Setting

## Abstract

Amidst competing priorities for allocating finite health resources, using evidence-informed priority setting is a valuable tool for achieving population-level health goals. The paper by Baltussen et al comprehensively reports on the development of practical guidance for evidence-informed deliberative processes (EDPs) which will help with sustainability of programs aimed at universal health coverage (UHC). The authors’ experience with the Joint Learning Network for UHC’s (JLN) peer-to-peer learning platform on evidence-informed priority setting offers insights on the practical challenges faced by countries in health benefits package (HBP) design, especially to draw in actors to advocate for the priorities and values across the health system. Lessons harvested from JLN countries that have established such advisory committees can provide practical insights for countries in earlier stages of establishing a systematic process for HBP design. Peer-to-peer learning modalities among countries offer viable and effective approaches to institutionalizing EDPs and systematic priority setting.

*Public revenues should be directed at health services and interventions that maximize progress toward universal health coverage (UHC)*. How can low- and middle-income countries (LMICs) do more with limited resources for health? Tackling key drivers of inefficiency — such as fragmentation of funding sources, vertical delivery systems, and gaps between investments and results — can help the health sector do more with limited resources. A major cause of health sector inefficiencies stems from decisions about resource allocation at the system, facility or physician level.^1^ In 2010, the World Health Organization (WHO) estimated that 20%-40% of healthcare expenditure was wasted, and inefficiency was a main driver of this wastage.^2^ More recently, it was estimated that in Organization for Economic Co-operation and Development countries, between 20% and 50% of health expenditures may be wasted due to inefficiencies.^2,3^ With this understanding, the Joint Learning Network for UHC (JLN) — a global network of practitioners and policy-makers sharing experiences about common challenges to develop and implement knowledge products supporting reforms for UHC, has focused on taking a systematic approach to understanding sources of and contextual factors leading to inefficiency, packaging findings into actionable policy recommendations and achieving efficacy through evidence-informed priority-setting and allocation of health spending.

*In the face of ever-increasing demand for healthcare and competing priorities, using evidence-informed priority setting to allocate finite resources is a valuable tool for achieving population-level health goals. *Evidence-informed priority setting occurs when decision-makers and the processes are explicit and transparent, and priority setting is done in a deliberative manner involving relevant stakeholders, in consideration of best available evidence about clinical and cost-effectiveness and social values.^4,5^ An evidence-informed health benefits package (HBP) — which defines the coverage of services, the proportion of the costs that are covered, and who can receive these services — is a powerful tool that can guide both the delivery of care and the associated resource allocation. A responsive, evidence-informed HBP is a dynamic policy instrument that is adjusted over time to address emerging challenges in implementation, changing disease burdens, fluctuating budgets, and the emergence of new services. However, a recent review of HBPs in LMICs shows that the majority of packages (14/24) have not been revised substantially – some despite having been in place for over a decade.^6^

 Health technology assessment (HTA) is a systematic process designed to inform priority setting and decision-making related to the integration of a technology or health intervention in to a HBP. This process can be used to develop and revise HBPs that enhance value for money and minimize opportunity costs associated with less impactful investments. HTA achieves this by facilitating the systematic prioritization of cost-effective health interventions.^1,7^

 The JLN has captured practical lessons from countries in conducting periodic revision of HBPs in a knowledge product titled, “Making Explicit Choices on the Path to UHC: Guide for Health Benefits Package Revision.”^8^ Guidance in this knowledge product can be used by practitioners in LMICs to support revision of their HBPs while responding to changing disease burdens, fluctuating budgets, and the emergence of new services and health technologies, and to correct for implementation challenges.

*Our experience with the JLN’s engagement in peer-to-peer learning can offer insights on the knowledge gaps at the country level that impact use of evidence-informed processes to determine the HBP design and implementation. *In Cambodia, an evidence-informed deliberative process (EDP) involving a group of key health sector stakeholders, which also included several non-health departments of the government as well as non-governmental entities, was facilitated using prioritization tools from the JLN. This helped inform the country’s prioritization of interventions that were included in the investment case for maternal, reproductive and child health. The consultative, multi-stakeholder process helped build consensus, acceptability and trust among the concerned stakeholders for the prioritized interventions.

 The paper on EDPs by Baltussen et al^9^ comprehensively reports on the development of practical guidance for EDPs. It outlines the processes, notably engaging input from stakeholders and the best available evidence that HTA bodies must follow to support legitimate HBP design. Baltussen et al EDP framework can also support the process of developing or revising an HBP while taking into account factors such as disease burden, equity issues, health system goals, and stakeholders’ preferences and values. This resonates very well with a JLN prioritization recommendation in situations of limited fiscal space, to target the poor with health services relevant for their needs. This approach optimizes the use of finite resources while remaining consisitent with societal values.

 National health insurance agencies (NHIAs), that tend to be the main users of the HTA agencies’ work, should have a buy-in into EDPs. However, research shows that in several LMICs, production of HTAs is disconnected from the process by which NHIAs make decisions about reimbursement. HTA methods guides in many JLN member countries do not even mention the NHIA. While such omission may not be deliberate, reasons behind it can vary as the process of priority setting can be very political. While a situational analysis that establishes why attempts to use data and evidence in the past have been successful or unsuccessful can shed light on how elements of the institutional environment and political economy might constrain or support the use of data and evidence in priority setting.^5^ In this context, EDPs offer a transparent and inclusive process by which legitimate, politically and technically sound recommendations can be made. In Indonesia, guidelines for HTA are being revised, with support from the JLN in helping Indonesia learn from its peers to include EDPs as part of the process, that will enhance credibility, legitimacy and eventual use of the HTA processes, especially by the NHIA.

 A foundational step of priority-setting is drawing in actors who can advocate for the priorities and values across all levels of the health system. Indeed, Baltussen et al^9^ advise that the first step in an EDP is installing an advisory committee that has a diverse and sufficient set of perspectives and technical skills, and can play a role in defining priorities. The advisory committee should have the criteria to assess those priorities, and to guide the process of selecting and appraising technologies and interventions through an HTA. One of the areas for practical implementation advice sought by countries and shared in the JLN’s publication on health priority setting^5^ is on setting up the right institutional mechanisms for EDPs. Lessons from JLN countries that have established such advisory committees — regarding their composition, role and mandate, and the criteria and guidelines used — can be a very useful insight for countries newer to these systematic processes. Such lessons, that can help smoothen the understanding of EDPs and their greater use by countries, could include: the mechanics of these deliberative processes- how to set these up; country learnings on practical aspects of setting up multiple levels of subcommittees if needed; modalities by which countries have provided opportunities to contribute to deliberations that do not depend only on physical participation in meetings – such as providing expert comments; country experiences and practices in compiling relevant evidence in a concise, accessible and standardized format, etc.

*Facilitating country understanding and use of EDPs can encourage periodical revisions of the benefit packages – and thereby the sustainability of health financing programs aimed at supporting progress toward universal health coverage.*If policy-makers have been postponing decisions regarding benefit package revisions due to the complexity of stakeholder dynamics and the potential for debates and resentment, sharing country experiences on the effectiveness of EDPs (Evidence-Based Packages) in fostering trust, credibility, and sustainability can promote their wider adoption. This, in turn, can encourage more frequent revisions of benefit packages. The value of peer learning for policy-makers is that this will help them understand the experiences with use of EDPs and how, if done well, they help build public trust in priority setting processes, and by extension the sustainability of UHC and health insurance programs. The failure to use EDPs in priority setting processes or to clearly communicate the application of such a process to the public can have implications such as patient or public resentment. Backlash against systematic priority setting processes can dampen use of such processes by national health bodies and NHIAs, introducing vulnerabilities to ad-hoc or discretionary decision making. When health budget allocations do not reflect health priorities or align with the prevailing policy and budget cycles, it becomes difficult to link expenditure plans with health objectives, and in turn to justify increases in resources for health. Additionally, when an HBP is underfunded, ill-defined, or has an infeasible scope of services, it can lead to inefficiencies and implicit rationing of health services, creating a ‘broken promise.’^10^ Given how EDPs offer a transparent and inclusive process to help undertake the necessary revisions in HBPs with legitimacy, we recommend advocacy for EDPs beyond the HTA agencies and the producers of the evidence, to add a focus on the main users of the HTA agencies’ work — the NHIAs, to understand and buy-in into EDPs, as part of their larger buy-in into both using evidence for designing benefit packages and systematic priority setting processes. This can strengthen the resourcing and resolve for using EDPs, and add an additional layer of accountability and transparency for EDPs.

*Peer-to-peer learning modalities among countries can offer a viable and effective mechanism to institutionalize EDPs as well as systematic priority setting itself. *Institutionalizing HTAs can be costly and technically challenging. Building upon the experience of LMICs in understanding the resource and capacity needs, as well as the possible sequencing of efforts, can be invaluable. One such example is the use of adaptive HTA processes in Indonesia during 2022 mentioned above, for which the country has received considerable catalytic inputs from peer countries, and was facilitated by the JLN and the international decision support initiative. As the importance of systematic priority setting (and embedded EDPs within these institutions) becomes better understood, many LMICs may consider introducing these processes. There is an opportunity for countries further along in the institutionalization of EDP to share lessons learned from their own experience, including emerging best practices and how challenges were successfully met.

*We recommend adding a research agenda to this framework, and using peer-to-peer learning to generate new, experience-based knowledge on the relevance of EDPs and how to undertake them better. *This is clearly an area for more exploratory work on a range of themes, which are amenable to both formal research as well as documentation of tacit knowledge held by country practitioners. Questions which could be further investigated include: Why do even established HTA agencies not have EDPs formally instituted? What factors enabled institutionalization of EDPs, especially in LMICs? What strategies have helped convince policy-makers about the importance of EDPs? Would identifying the consequences of decision-making in the absence of an EDP support advocacy for the adoption of EDPs? To better understand how EDPs can be advocated for and institutionalized, it is useful to understand what specifically poses a challenge in setting up and operationalizing EDPs, and what enables it. Exchanges and interactions between practitioners in peer countries can be a valuable approach to capturing experiential knowledge as a complementary modality to formal research, and inspiring and catalyzing policy-makers by distilling knowledge from the successful experiences of their peers.

## Acknowledgements

 The authors would like to thank the facilitators and members of the JLN Efficiency collaborative for the content and examples used in this paper.

## Ethical issues

 Not applicable.

## Competing interests

 Authors declare that they have no competing interests.

## Funding

 There was no funding received specifically for this paper. This work was supported by respective organizations to which the individual authors are affiliated, to the extent that their time was covered by their employers, where applicable.
